# A32 DEVELOPMENT AND VALIDATION OF THE TORONTO UPPER GASTROINTESTINAL CLEANING SCORE

**DOI:** 10.1093/jcag/gwab049.031

**Published:** 2022-02-21

**Authors:** S Seleq, R Khan, N Gimpaya, J I Vargas, S Amin, M Bilal, S Bollipo, A Charabaty, E de-Madaria, A Hashim, J Kral, K M Pawlak, D S Sandhu, R N Lui, S Sanchez-Luna, K Siau, J Mosko, S Grover

**Affiliations:** 1 Department of Medicine, University of Toronto, Toronto, ON, Canada; 2 Pontificia Universidad Catolica de Chile, Santiago, Chile; 3 Division of Digestive Health and Liver Diseases, Department of Medicine, University of Miami Miller School of Medicine, Miami, Florida, Miami, FL; 4 Division of Gastroenterology & Hepatology, Beth Israel Deaconess Medical Center, Boston, Massachusetts, Boston, MA; 5 Gastroenterology Department, John Hunter Hospital, University of Newcastle, Newcastle, New South Wales, Australia, Newcastle, New South Wales, Australia; 6 Division of Gastroenterology, Johns Hopkins-Sibley Memorial Hospital, Washington, DC, Washington, DC; 7 Alicante University General Hospital, Alicante Institute for Health and Biomedical Research, Alicante, Spain, Alicante, Spain; 8 Institution for Clinical and Experimental Medicine, Prague, Czech Republic, Prague, Czechia; 9 Hospital of the Ministry of Interior and Administration, Szczecin, Poland, Szczecin, Poland; 10 Division of Gastroenterology, Hepatology & Nutrition, Cleveland Clinic, Cleveland, Ohio, Cleveland, OH; 11 Division of Gastroenterology and Hepatology, Institute of Digestive Disease, The Chinese University of Hong Kong, Hong Kong, China, Hong Kong, China; 12 Institute of Translational Medicine, University Hospitals Birmingham, Birmingham, Birmingham, United Kingdom; 13 Department of Medicine, University of Jeddah, Jeddah, Saudi Arabia; 14 Division of Gastroenterology and Hepatology, The University of New Mexico, Albuquerque, NM; 15 St Michael’s Hospital, Toronto, ON, Canada

## Abstract

**Background:**

High quality esophagogastroduodenoscopy (EGD) depends on the ability to appropriately visualize upper gastrointestinal (GI) mucosa pathology. Evaluation can be limited by the presence of mucus, foam, bubbles and solid materials. Currently, there is no standardized method to assess mucosal visualization for use in clinical or research settings.

**Aims:**

To develop and establish the content validity of the Toronto Upper Gastrointestinal Cleaning Score (TUGCS) and evaluate its interrater reliability.

**Methods:**

An international panel of endoscopy experts rated potential items and their associated anchors for importance as indicators of adequacy of mucosal visualization during EGD. The survey utilized a Likert scale (1 (strongly disagree) to 5 (strongly agree)). The Delphi process was repeated until consensus was reached. Consensus was defined priori as ≥80% of experts in a given round scoring ≥4 on all survey items. To assess content validity, 48 EGD procedures were evaluated in real-time by two endoscopist reviewers using the TUGCS at a single institution. The interrater agreement between assessments was calculated for TUGCS total scores using intraclass correlation coefficient, one-way random effects model (ICC 1,1).

**Results:**

Fourteen experts agreed to be part of the Delphi panel. An anatomical framework representing the upper GI mucosa and anchors for each mucosal portion representing various levels of visibility was generated through systematic review. Three survey rounds, with response rates of 100%, 100% and 71% respectively, achieved consensus. The final TUGCS includes four anatomical areas (fundus, body, antrum, duodenum) and mucosal visualization anchors ranging from 0 to 3 (Figure 1). TUGCS was used to assess foregut cleaning in 48 procedures (Table 1). The mean TUGCS for staff and trainee were 8.1 (±2.4) and 8.1 (±2.6), respectively. The ICC was 0.78 (95% confidence interval 0.62–0.88) indicating good reliability.

**Conclusions:**

We developed and generated content validity evidence for the TUGCS through rigorous Delphi methodology, reflective of practice across different centres. Planned as future research is a video survey distributed to endoscopists internationally to further validate the TUGCS to create a tool that may be used to judge mucosal visualization for EGD in research and clinical settings.

**Funding Agencies:**

None

**Table:** Table 1. Patient Characteristics (n=48)

Age, mean (±SD)		55.4 (18.6)
Sex, n		
	Male	20
Female	28
Fasting duration (hours), mean (±SD)		14.9 (10.5)
Procedure type		
	Elective	47
Acute	1

**Figure:**
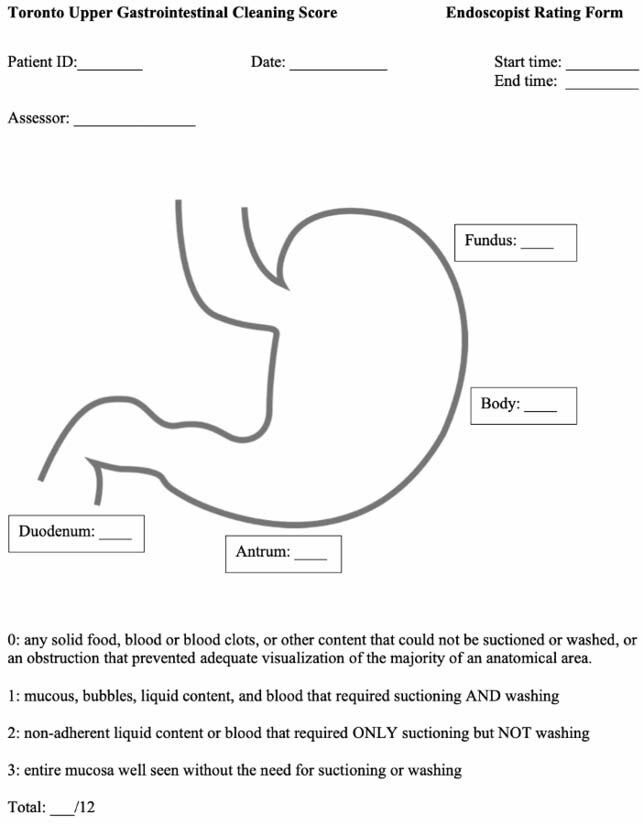
Figure 1. TUGCS tool

